# Prevalence and Etiopathogenic Profile of Oral Squamous Cell Carcinoma in Nonsmokers and Nondrinkers: Expanding Risk Determinants Beyond Tobacco Exposure

**DOI:** 10.3390/diagnostics16101563

**Published:** 2026-05-21

**Authors:** Effimia Stergiadou, Alexandros Louizakis, Dimitris Tatsis, Asterios Antoniou, Konstantinos Poulopoulos, Athanasios Poulopoulos

**Affiliations:** 1 Department of Oral Medicine and Pathology, School of Dentistry, Aristotle University of Thessaloniki, 54124 Thessaloniki, Greece; stergiefi@gmail.com (E.S.); akpoul@dent.auth.gr (A.P.); 2 Department of Oral and Maxillofacial Surgery, Aristotle University of Thessaloniki, 54124 Thessaloniki, Greece; dimitats@auth.gr (D.T.); ast.antoniou@gmail.com (A.A.); 3 Division of Oral Surgery, Implantology and Roentgenology, Aristotle University of Thessaloniki, 54124 Thessaloniki, Greece; kpoulopa@dent.auth.gr

**Keywords:** chronic mechanical irritation, female predominance, nonsmoking nondrinking oral squamous cell carcinoma, oral microbiome, tongue cancer

## Abstract

Oral squamous cell carcinoma (OSCC), comprising ~90% of oral malignancies, remains a major global health burden with rising incidence despite declining tobacco use. While tobacco and alcohol are classic dominant risk factors, a distinct subgroup of nonsmoking, nondrinking (NSND) patients is increasingly recognized, accounting for 15–35% of OSCC cases in many cohorts, particularly in developed countries. This emerging epidemic shows striking demographic patterns: strong female predominance (often 65–77% of cases), bimodal age distribution with peaks in young adults (<45 years) and elderly individuals (>70 years), and overrepresentation among non-Hispanic White and certain Asian populations. Unlike traditional habit-related OSCC, which favors the floor of the mouth in older males, NSND tumors predominantly arise on the lateral tongue, gingiva, and buccal mucosa. Etiopathogenesis extends far beyond conventional carcinogens and involves multifactorial mechanisms, including chronic mechanical irritation from dental factors, oral microbiome dysbiosis enriched with periodontal pathogens (e.g., *Fusobacterium nucleatum* and *Porphyromonas gingivalis*), limited roles for viruses (minimal HPV contribution, possible EBV or “hit-and-run” HSV effects), genetic susceptibilities (e.g., Fanconi anemia and CDKN2A mutations), epigenetic changes, hormonal influences contributing to female bias, metabolic conditions (diabetes and hyperlipidemia), poor oral hygiene, and chronic inflammation. NSND OSCC frequently exhibits a distinct immunological profile with higher tumor-infiltrating lymphocytes and PD-L1 expression, potentially favoring immunotherapy, though prognosis remains heterogeneous—better in some cohorts due to fewer comorbidities, yet worse in young patients with higher recurrence and second primary tumor risks. Delayed diagnosis is common due to low suspicion in “low-risk” individuals. This review underscores NSND OSCC as a unique entity requiring expanded risk assessment, heightened clinical vigilance for persistent oral lesions regardless of habit history, and targeted research into novel prevention and therapeutic strategies.

## 1. Introduction

Oral squamous cell carcinoma (OSCC) is the most prevalent cancer of the oral cavity and constitutes a major health problem worldwide. It has classically been closely linked to well-documented risk factors, most notably tobacco usage, alcohol intake and, in some places, betel quid and areca nut use [1,2,3].

However, in recent decades, a substantial epidemiological transition has been documented. Although tobacco consumption is declining worldwide, the prevalence of OSCC is still increasing, pointing to alternative carcinogenic pathways [4,5,6,7]. Cases are increasingly being seen in patients without a history of smoking and alcohol use, known as nonsmoking, nondrinking (NSND) patients, who account for roughly 10–35% of OSCC in recent cohorts [8,9].

This new subgroup presents different demographic and clinical characteristics. NSND OSCC is more common in women and has a bimodal age distribution, occurring in both younger and older persons [8,9,10]. In addition, tumor localisation patterns are different from conventional cases, favoring the lateral tongue, gingiva and buccal mucosa instead of the floor of the mouth [2,8]. Such differences show that NSND OSCC is more than a lack of established risk factors, but rather may represent a biologically unique entity.

The etiopathogenesis of NSND OSCC is complex and still not fully explained. Possible contributing factors include prolonged mechanical irritation, dysbiosis of the oral microbiota, genetic vulnerability, metabolic and hormonal effects, and immune-mediated pathways [3,11,12,13,14]. Moreover, the absence of classical risk factors typically leads to an overestimate of the cancer risk in these individuals, thereby favoring a delayed diagnosis and possibly worse clinical results [15,16].

Our results underline the need to move beyond usual models of OSCC etiology and to properly characterize this expanding population of patients. This is a narrative review that examines the emerging epidemic of oral squamous cell carcinoma (OSCC) in the nonsmoking, nondrinking (NSND) population by addressing two primary objectives: describing its distinct prevalence patterns and analyzing its complex etiopathogenic profile beyond traditional tobacco and alcohol exposure.

### 1.1. Definition of the NSND Population

For the purpose of this review, the term “nonsmoking, nondrinking (NSND)” individuals is defined as patients who do not currently or previously have a history of habitual use of tobacco and who do not regularly consume alcohol. However, research differs in the definitions of “nonsmoker” and “nondrinker,” with some studies including former smokers, occasional alcohol users, or those with incomplete exposure histories. This diversity can alter reported prevalence, demographic characteristics, and relationships with risk factors and should be addressed when interpreting the data.

### 1.2. Search Strategy

This review was conducted by searching major electronic databases, including PubMed/MEDLINE, Google Scholar and Scopus, for relevant English-language articles that were published between 2008 and 2026. The key search terms that were used included combinations of “oral squamous cell carcinoma” OR “OSCC” AND “nonsmoker*” OR “non-smoker*” OR “never smoker” OR “nondrinker*” OR “non-drinker*” OR “NSND” AND “risk factors” OR “etiology” OR “prevalence” OR “epidemiology” OR “pathogenesis”. Additional filters were added for reviews, clinical studies, and epidemiological data focused on NSND cohorts. Article selection prioritized peer-reviewed studies addressing demographic features, prevalence patterns and also alternative etiopathogenic mechanisms in NSND patients, resulting in 47 key references that were included in the final synthesis. Only studies published in English were considered. Due to heterogeneity in study design and definitions of NSND populations, a qualitative synthesis approach was adopted rather than a formal meta-analysis. For the purposes of this review, NSND refers to individuals with no current or prior habitual tobacco use and no regular alcohol consumption, acknowledging that definitions may vary across studies.

## 2. Prevalence of OSCC in Nonsmokers and Nondrinkers

While tobacco and alcohol are the primary drivers of OSCC globally, patients with no history of these habits—the nonsmoking, nondrinking (NSND) cohort—constitute a growing and distinct subgroup. NSND individuals represent approximately 10% to 15% of all head and neck squamous cell carcinomas worldwide, but they account for a much larger proportion—between 15% and 35%—of the isolated OSCC population [1,2].

### 2.1. Regional and Geographic Variations

There are specific regional and geographic variations in these prevalence rates:

- Developed nations: In countries with declining tobacco use, the proportion of NSND OSCC patients is remarkably high. OSCC-specific studies report NSND prevalence rates of 32.4% in the Netherlands [1], 33.1% among patients over 45 years in Japan [8] and similarly, high rates ranging from 24% to 35% in Australian and American cohorts [17].
- In contrast, in regions such as the Indian subcontinent, where the use of smokeless tobacco, betel quid, and areca nut is endemic, nonhabit-related OSCC is relatively rare, accounting for only 4% to 6% of oral cancer cases [11].

A defining demographic feature of the NSND OSCC cohort is a strong female predilection. While traditional habit-associated OSCC is twice as likely to affect men, NSND OSCC patients are two to three times more likely to be female [12,17]. Across various global cohorts, women constitute the majority of the NSND group, frequently accounting for 65% to 76.9% of these patients [1,8]. Furthermore, the increasing incidence of OSCC over the past decades has been most prominent among young women without traditional risk factors [8].

### 2.2. Age Distribution Characteristics

In addition, unlike traditional OSCC, which typically peaks in men in their sixth and seventh decades, NSND OSCC appears to have a bimodal age distribution, significantly affecting both young and elderly individuals [17].

- Young adults (under 40–45 years): There is a rising incidence of OSCC among young adults, with studies showing that up to 41% of NSND patients are younger than 50 years [18]. In patients under 45 years of age, tongue cancer is the predominant manifestation, accounting for more than 90% of OSCC diagnoses in this age group, many of whom lack traditional risk factors [8].
- Elderly individuals (over 70 years): The second peak occurs in elderly individuals, particularly women. One Japanese multicenter study reported that the median age of NSND patients was 75 years, with women aged 70–84 accounting for the vast majority of the cohort [8]. Similarly, another cohort reported that 64.2% of NSND patients were over 70 years old [2].

### 2.3. Ethnicity Patterns

Data on ethnicity in NSND OSCC patients are predominantly derived from United States registries. Analyses of the Surveillance, Epidemiology, and End Results (SEER) database indicate that the increasing incidence of OSCC in young patients without traditional risk factors is occurring almost exclusively among non-Hispanic White individuals, with the steepest increase observed in young White women [12]. Additionally, one large US-based epidemiological study revealed that Asian Americans were overrepresented in the NSND OSCC group compared with the smoking and drinking cohort, which had a greater representation of African Americans [18].

### 2.4. Socioeconomic Status Considerations

While direct causal links between socioeconomic status (SES) and NSND OSCC are still being explored, SES serves as a significant demographic denominator, primarily because of its strong association with tobacco and alcohol consumption. Generally, lower socioeconomic status and lower education levels are strongly associated with higher rates of smoking and alcohol abuse and, consequently, traditional habit-associated OSCC [9,10]. Studies have shown a correlation in which patients with higher socioeconomic and occupational statuses are less likely to smoke heavily, which indirectly concentrates NSND OSCC cases among populations with better socioeconomic standing and access to care [9]. Table 1 summarizes core comparisons between traditional and NSND oral cancer groups.

## 3. Etiopathogenic Profile: Expanding Risk Determinants

The etiopathogenesis of oral squamous cell carcinoma (OSCC) in nonsmokers and nondrinkers (NSND) is increasingly acknowledged as a distinct clinical and molecular entity [27]. Although established risk factors such as tobacco and alcohol consumption account for approximately 80% of cases, an increasing incidence is observed among individuals without these habits, especially younger women. This trend necessitates a broader investigation of etiological factors beyond classical chemical carcinogens [4,11]. This pathogenic profile is defined by a complex interaction between infectious, genetic, environmental, and immunological factors.

### 3.1. Infectious Contributors

The involvement of viruses in oropharyngeal squamous cell carcinoma (OPSCC) has been well described. However, their role in OSCC is not yet fully understood and is still under investigation. Although human papillomavirus (HPV), particularly HPV-16, is a well-known etiological factor of OPSCC, its role in NSND patients with OSCC is significantly limited [3,19]. While HPV is prevalent in 70% of OPSCC cases, its prevalence in OSCC cases is much lower, ranging from 2.6% to 5.9% [5]. Furthermore, p16 overexpression is a poor predictor of HPV-driven oncogenesis in the oral cavity, as HPV at this subsite exhibits minimal transcriptional activity [6]. Additionally, Epstein–Barr virus (EBV) has been detected in OSCC tissues and may contribute to oncogenesis through oncoproteins such as LMP-1 [7,10]. In addition, a new mechanism called “hit and run” has been hypothesized for viruses such as herpes simplex virus type 2 (HSV-2). This mechanism suggests that self-limited infection triggers epigenetic deregulation that persists after viral clearance and could trigger cancer development in individuals without known risk habits [3,15]. While HPV has a clearly limited role in NSND OSCC with low prevalence and minimal transcriptional activity, the potential contributions of EBV (via LMP-1) and HSV-2 (through a “hit-and-run” mechanism) remain suggestive in this population; however, the overall strength of evidence is moderate and requires further validation in larger NSND-specific studies [3,15].

### 3.2. Genetic and Molecular Factors

Hereditary factors play crucial roles in OSCC development. This is especially evident in patients with Fanconi anemia (FA), where impaired DNA repair mechanisms result in a 500- to 800-fold increased risk of OSCC, which frequently occurs in young adults [11]. Alterations in detoxification-related genes, including null genotypes of GSTM1 and GSTT1 and CYP1A1 variants, may impair the body’s ability to metabolize environmental carcinogens, leading to increased vulnerability in affected individuals [1,28]. Epigenetic alterations, specifically DNA hypermethylation of CpG islands and silencing of tumor suppressor genes such as CDKN2A, p16, and p15, are critical in the development of oral cancer in patients without known behavioral risk factors (Pandiar and Krishnan et al. (2024) [29]; Roman et al. (2021) [5]). In NSND patients with OSCC, somatic mutations are predominantly observed in CDKN2A, with frequent EGFR amplifications and BRCA2 deletions, whereas tobacco-related tumors are characterized mainly by TP53 alterations [22,30]. However, current evidence indicates that the molecular profile of NSND OSCC is more heterogeneous than previously emphasized, encompassing diverse genetic and epigenetic alterations that differ from the predominant TP53 mutations observed in tobacco-associated OSCC [3,30].

### 3.3. Environmental and Lifestyle Influences

Despite common lifestyle-related etiological factors, exposure to carcinogenic air pollutants, such as particulate matter and chemical irritants, is also an established risk factor for OSCC [1]. Furthermore, exposure to occupational hazards in industries such as rubber, paint, and pesticide production is also linked to OSCC development in nonhabit patients [16,18]. In addition, diet plays an important role in OSCC; more specifically, insufficient consumption of fruits and vegetables rich in antioxidants represents a significant dietary risk, contributing to approximately 11–15% of oral cancers worldwide [11,25]. Additionally, poor oral hygiene remains a significant etiological factor, as low tooth-brushing frequency, tooth loss, and irregular dental consultations are intricately connected to increased risk for OSCC and poorer survival rates [4,20]. Among these exposures, poor oral hygiene and chronic mechanical irritation emerge as particularly relevant and strongly associated factors in NSND populations, likely acting through sustained local inflammation and oxidative stress, whereas the contributions of air pollution, occupational risks, and dietary factors appear less significant and require more precise evaluation in NSND-specific studies [5,9].

### 3.4. Immunological and Inflammatory Mechanisms

Chronic inflammation is a well-established contributing factor in the development of oral cancer among NSND individuals. Recent research has demonstrated that periodontal disease is independently linked to an approximately fivefold increased risk of OSCC in these patients [4]. Systemic immunosuppression resulting from HIV infection, autoimmune disorders, or therapeutic interventions significantly increases the risk of malignant transformation [4,11]. The abovementioned inflammatory and immune-altering conditions are intricately connected to microbiome changes that cause dysbiosis in the oral cavity. In NSND patients, pathogenic species such as *Fusobacterium nucleatum*, *Porphyromonas gingivalis*, and *Slackia exigua* are enriched and actively contribute to a proinflammatory and genotoxic tumor microenvironment [6,7]. Another important contributing factor of OSCC in NSND individuals is oral potentially malignant disorders (OPMDs), especially oral lichen planus [19]. Chronic inflammation and microbiome-induced immune modulation create a tumor-permissive microenvironment in NSND OSCC, characterized by increased tumor-infiltrating lymphocytes (TILs) and upregulated PD-L1 expression. This unique immunological profile not only promotes carcinogenesis but may also indicate enhanced responsiveness to immune checkpoint inhibitors compared with conventional tobacco-related OSCC [27].

### 3.5. Emerging and Multifactorial Risks

Recent studies have shown that specific hormonal factors, such as abnormal estradiol metabolism and pituitary hormone imbalances, are possible contributors to the female predominance observed in NSND patients with OSCC [23,31]. Moreover, metabolic syndromes, such as diabetes mellitus and hyperlipidemia, are also recognized as significant independent risk factors [4,20]. In addition, chronic mechanical irritation in the oral cavity caused by fractured restorations, sharp teeth, or ill-fitting dentures has been proposed as a synergistic factor in oral carcinogenesis. Oxidative DNA damage may be promoted through repeated injury–repair cycles, leading to the activation of oncogenes [9,29]. Although associations with hormonal factors, metabolic syndromes, and chronic mechanical irritation have been reported in NSND OSCC, the current evidence remains largely observational and is derived from retrospective studies with inherent limitations. These factors likely act synergistically with chronic inflammation and microbiome dysbiosis, but further prospective studies are needed to establish their independent contribution and mechanistic relevance in this population [3].

### 3.6. Microbiology

Researchers have utilized recent advances in third-generation sequencing (TGS) to identify distinct microbial signatures uniquely associated with habit-free populations [7]. High accuracy in discriminating tumor tissue in habit-free patients has been demonstrated by a three-species panel consisting of *Eikenella corrodens*, *Slackia exigua*, and *Eggerthia catenaformis* [7]. Carcinogenesis is facilitated by these species through proinflammatory metabolic pathways, including styrene degradation and alterations in lipid and glutamine metabolism, whereas the transition to a malignant state is characterized by the loss of commensal taxa such as *Prevotella pallens* and *Streptococcus oralis* [3,7]. Although these microbial signatures show promising ability to distinguish tumor from normal tissue, the findings are currently based on limited cohorts and require validation in larger, independent studies to confirm their robustness and reproducibility. While these taxa are strongly associated with NSND OSCC and may contribute to carcinogenesis via chronic inflammation and genotoxic metabolite production, current evidence remains largely correlative and longitudinal studies are essential to establish causality and clarify their precise biological role in oral oncogenesis [7]. Table 2 summarizes the main differences between traditional tobacco- and alcohol-related OSCC and NSND OSCC with respect to demographics, tumor site, prevalence trends, etiologic profile, and clinical implications.

**Figure 1:**
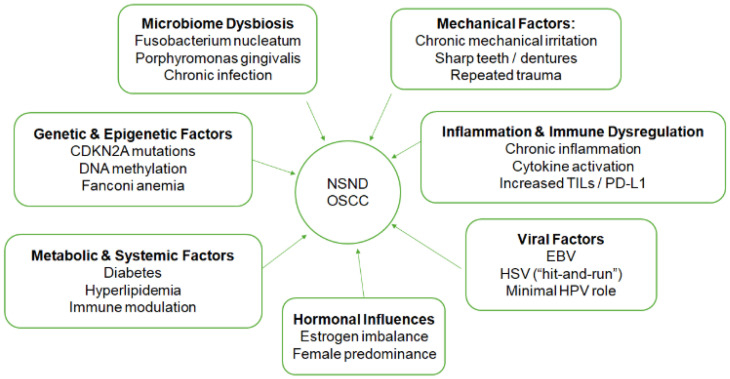
Etiopathogenic pathways for oral squamous cell carcinoma in nonsmokers and nondrinkers.

Figure 1 shows the multifactorial etiopathogenic pathways proposed for oral squamous cell carcinoma in nonsmokers and nondrinkers (NSND OSCC). NSND OSCC is a consequence of a complicated interplay of several atypical risk factors. Chronic mechanical irritation, dysbiosis of the microbiome and persistent inflammation all contribute to a pro-carcinogenic microenvironment. In the absence of typical carcinogens like smoke and alcohol, malignant transformation is further driven by genetic and epigenetic changes and metabolic, hormonal and possibly viral factors.

## 4. Clinical and Pathological Characteristics

### 4.1. Clinical and Pathological Features

**Figure 2:**
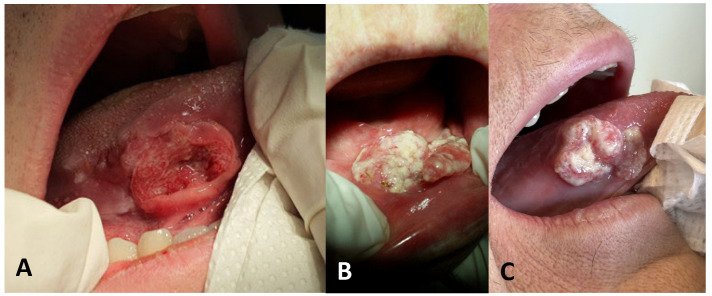
Clinical features of nonsmoking, nondrinking patients with oral cancer. (**A**) Ulceration on the lateral border of the tongue in a 22-year-old male patient. (**B**) Exophytic lesion on the floor of the mouth in a 79-year-old female patient. (**C**) Wide exophytic lesion on the lateral border of the tongue in a 70-year-old female patient.

The anatomical distribution of tumors in nonsmokers is substantially different from that in smokers in terms of site preferences. The tongue, especially the lateral border, is the most frequently occurring primary site [6]. Over 85% to 90% of cases in some cohorts of young nonsmokers are tongue-related cancers (Figure 2) [1,8]. In contrast to smokers, nonsmokers exhibit a greater preference for the maxillary and mandibular alveolar ridges and buccal mucosa [2,19,20]. Last, it is quite uncommon for nonsmokers to have floor-of-mouth involvement [2,19,20]. Carcinogens are believed to remain on the floor of the mouth with saliva in smokers, whereas this area is largely shielded in nonsmokers [16,17].

### 4.2. Histopathological Features

In terms of differentiation and keratinization, the majority of tumors in this category are of the keratinizing type in nature [6]. They are often scored similarly or with a considerable degree of differentiation [16]. In terms of the invasion pattern, some studies indicate less aggressive characteristics, such as lower rates of desmoplastic stroma and perineural invasion (PNI), than smokers do, whereas other studies indicate that younger patients may have high rates of tumor budding and aggressive patterns of invasion (POI 3 or 4), which are associated with poorer survival [6,27]. Additionally, a unique immune-modulated phenotype frequently characterizes the immunological microenvironment for OSCC in nonsmokers [19]. Higher quantities of tumor-infiltrating lymphocytes (TILs) and markedly increased PD-1 and PD-L1 expressions are examples of this, indicating that they would be better candidates for immune checkpoint therapy [1,14,15].

### 4.3. Treatment Response, Prognosis and Survival

There is ongoing discussion in the literature on the prognosis of OSCC in nonsmokers in comparison with conventional cases. Nonsmokers have increased overall survival (OS) and disease-specific survival (DSS) rates, according to numerous large-scale studies [3,4,27]. This is frequently linked to a less aggressive tumor phenotype and fewer medical comorbidities [12]. In contrast to older patients or young smokers, a number of writers have documented worse results, particularly for young nonsmokers [6,22]. This has led to the hypothesis that young-onset OSCC may be driven by distinct genetic predispositions (such as Fanconi anemia) or unique molecular drivers that cause more aggressive behavior [3,6,11].

Despite having fewer comorbidities and lower invasion depths, young nonsmokers frequently receive more aggressive treatment, including higher rates of adjuvant chemoradiation [12,17]. With respect to recurrence, some reports indicate that nonsmokers, particularly elderly females, may face higher rates of tumor recurrence and a significantly increased risk (up to 3.9-fold) of developing second primary tumors [1,17,24,32]. Another explanation for the development of a second primary disease is that NSND patients are typically young, and there is a prolonged time for de novo mutations that can cause a new cancer.

Although Fanconi anemia contributes to aggressive disease in a small subset of young NSND patients, its overall impact is limited due to its rarity. Broader drivers, including distinct molecular subtypes, enhanced immune profiles with higher tumor-infiltrating lymphocytes (TILs) and PD-L1 expression, microbiome alterations, and chronic inflammation, likely play more substantial roles in the heterogeneous prognosis of NSND OSCC [3,27]. Conflicting survival outcomes are strongly influenced by age and sex, with elderly NSND females generally demonstrating better survival due to fewer comorbidities, while some young patients experience higher recurrence rates [8,12]. The more aggressive treatment in younger patients is typically justified by their longer life expectancy and elevated risk of recurrence and second primary tumors. This increased risk of second primary tumors in NSND patients is likely attributable to both underlying biological susceptibility (genetic instability and chronic inflammation) and longer follow-up time resulting from improved overall survival [1,17].

### 4.4. Diagnostic Challenges and Delayed Detection

Delayed detection is crucial in the clinical care of nonsmokers. The diagnosis of OSCC is often overlooked in its early stages since these patients do not fit the “traditional” risk profile. The risk of cancer in young, healthy nonsmokers is commonly underestimated by both patients and healthcare professionals [13,19]. Initial symptoms, such as nonhealing ulcers, are often misinterpreted or ignored [29]. Additionally, persistent mucosal irritation caused by sharp teeth, ill-fitting dentures, or dental gear is thought to be an etiologic trigger among nonhabit users [9,16,23]. Even in the absence of a history of alcohol or tobacco use, this frequently leads to lesions on the lateral border of the tongue, which should be addressed with a high index of suspicion (Mehmi et al., 2022 [16]; Neckel et al. (2020) [13]). Finally, nonsmokers may present potentially malignant oral disorders (OPMDs), such as leukoplakia or oral lichen planus, which have been demonstrated to have a greater risk of malignant transformation in this population than smokers do [19,27]. In conclusion, OSCC in nonsmokers is a distinctive clinical entity characterized by particular site predilections and a unique immunological profile. Even if these individuals do not have any of the usual risk factors, they nonetheless need careful screening and long-term monitoring because of the dangers of delayed diagnosis and recurrence [13]. This is especially true for young and old people.

## 5. Discussion

The collective evidence from the sources establishes oral squamous cell carcinoma (OSCC) in nonsmoking and nondrinking (NSND) patients as a distinct clinico-pathological and biological entity rather than merely a subgroup defined by the absence of the traditional habits [8,25]. This population represents a significant and potentially increasing proportion of total OSCC cases [8].

The demographic profile of NSND patients is characterized by a strong female predominance and a bimodal age distribution, with clusters in young adults (often <40 or 45 years) and elderly individuals [10,24]. While Harada et al. (2023) [10] identified “young tongue” and “elderly female” as the two primary subgroups, Judd et al. (2025) [12] found that young NSND patients not only present with fewer medical comorbidities but also with a lower depth of invasion compared to older smoker–drinker (SD) patients.

Anatomic subsite predilections further differentiate this group. NSND tumors are mostly found on the oral tongue, buccal mucosa and maxillary alveolus [10,11]. Conversely, SD cohorts show a high predilection for the retromolar trigone and the floor of the mouth (FOM). Lalremtluangi et al. (2024) and Mehmi et al. (2022) observed that FOM involvement is more than 26 times more often associated with traditional risk factors, likely because the thin, non-keratinized mucosa in this area allows for the pooling of carcinogens in saliva [11,16].

To continue, several alternative mechanisms have been proposed in the absence of chemical carcinogens. Chronic mechanical irritation (CMI), namely trauma from sharp teeth, ill-fitting dentures, or dental hardware, is a significant factor in habit-free carcinogenesis [22]. Pandiar and Krishnan (2024) highlight that the lateral border of the tongue is particularly susceptible to trauma-induced malignancy, where chronic injury–repair cycles may trigger malignant transformation through mechanisms like chromoanagenesis [29].

Regarding the microbiome and viruses, the role of the human papillomavirus (HPV) in the oral cavity remains limited, although it is a major driver in the oropharynx, with most habit-free OSCC cases testing negative [6]. Instead, Foy et al. (2020) suggest a potential viral origin involving viruses like HSV-1 via a “hit-and-run” mechanism that triggers epigenetic deregulation [15]. Additionally, Rahman et al. (2022) and Yang et al. (2021) identified specific microbial biomarkers, such as periodontal pathogens, that may modulate the tumor microenvironment in habit-free individuals [4,7].

In terms of genetics and systemic health, Koo et al. (2021) identified unique genomic signatures in NSND patients, including enrichment of CDKN2A mutations and EGFR amplifications [28]. Furthermore, hereditary syndromes like Fanconi anemia significantly predispose individuals to early-onset OSCC [3,11]. Yang et al. (2021) also identified systemic factors such as eating disorders, depression, and HIV infection as independent predictors of OSCC in never-smoking adults [4].

The survival outcomes of NSND patients versus SD patients remain controversial. NSND status was associated with improved survival and a stronger immunogenic microenvironment with greater tumor-infiltrating lymphocytes (TILs) and increased PD-1/PD-L1 expression, as shown by Fiedler et al. (2025) [14]. Yang et al. (2021) observed considerably higher disease-specific survival in NSND patients [4]. Similarly, Judd et al. [12] have suggested that after adjustment for stage and comorbidities, survival rates may be comparable.

A critical finding by Johnson et al. (2020) is that NSND patients face a significantly higher risk—estimated at 3.9-fold—of developing second primary tumors (SPTs) [1]. This suggests a mechanism of field cancerization that is independent of tobacco and alcohol, necessitating rigorous long-term surveillance for this [10]. Risk stratification for NSND patients has improved with innovations in maxillofacial surgery, including the introduction of depth of invasion (DOI) in the 8th edition of the AJCC staging system [11,17]. The immunogenic profile of these tumors suggests they may derive greater clinical benefit from immune checkpoint inhibitors like pembrolizumab [14]. These findings underscore the need for a multidisciplinary and personalized approach that accounts for the unique biological behavior and diagnostic challenges inherent to OSCC in patients without traditional risk factors [8].

The recognition of nonsmoking, nondrinking (NSND) oral squamous cell carcinoma as a distinct clinical entity has important translational implications [14,19,24]. Accurate risk assessment should not be limited to traditional risk variables alone, as limiting it to tobacco and alcohol exposure may lead to underdiagnosis in this population [13,16]. Stratification of patients based on demographic, clinical and molecular factors is important, since NSND cases may differ in presentation, biological behavior and prognosis, and hence influence diagnostic techniques and treatment planning [8,22,24]. Furthermore, the higher probability of delayed diagnosis, the risk of recurrence and second primary tumors indicate the need for diligent monitoring and long-term follow-up [1,11,12,26]. Patient education is particularly important to promote knowledge that oral cancer can arise even in the absence of conventional risk factors, promoting faster evaluation of suspicious lesions [13,16].

## 6. Strengths and Limitations

This review underscores the need to recognize the nonsmoking and nondrinking oral squamous cell carcinoma (NSND OSCC) as a distinct clinical entity as well as to highlight growing evidence for unconventional risk factors, namely microbiome dysbiosis, chronic mechanical irritation, and immune-related mechanisms. However, the current literature is limited by heterogeneity in study design, population characteristics, and inconsistent definitions of NSND status, which restricts comparability and generalizability. The fact that the majority of our research is mostly based on retrospective and observational data further limits our research. Moreover, the narrative methodology, although it is appropriate for integrating heterogeneous evidence, does not permit quantitative synthesis. These limitations emphasize the need for standardized NSND classifications and well-designed, prospective multicenter studies. Future research should prioritize multi-omics approaches to better emphasize the underlying pathophysiology and also support the development of early detection strategies, robust risk stratification models and targeted therapeutic interventions, particularly immunomodulatory approaches, in order to improve clinical outcomes in this expanding patient population.

## 7. Future Directions

Future directions in research on nonsmoking, nondrinking oral squamous cell carcinoma should prioritize elucidating its multifactorial etiology through large-scale, prospective multicenter studies that integrate multi-omics approaches, including detailed tumor mutational profiling (e.g., next-generation sequencing for CDKN2A, EGFR, and BRCA2 alterations), microbiome sequencing, and immune phenotyping of the tumor microenvironment. Investigating the role of chronic mechanical irritation, oral dysbiosis with periodontal pathogens, epigenetic changes, hormonal influences (particularly in females), and immune-modulating comorbidities will help clarify distinct carcinogenic pathways. Developing standardized definitions and risk stratification tools for NSND patients is essential, alongside exploring saliva-based microbial biomarkers for early detection and screening. Given the observed immune-modulated phenotype (e.g., higher PD-L1 expression and tumor-infiltrating lymphocytes), trials evaluating immune checkpoint inhibitors and immunopreventive strategies hold promise for tailored therapies. Longitudinal studies addressing the elevated risk of second primary tumors and recurrence, especially in young and elderly subgroups, are needed to optimize surveillance protocols and improve outcomes in this growing, clinically distinct population.

## 8. Conclusions

Oral squamous cell carcinoma in nonsmokers and nondrinkers (NSND) is an increasingly recognized and clinically separate subpopulation with different demographic, anatomical and biological traits compared to classic tobacco- and alcohol-linked illness. Its multiple pathophysiology is thought to include chronic inflammation, mechanical irritation, microbiome dysbiosis, genetic vulnerability, metabolic effects and immunologically linked pathways. Often, these patients lack conventional risk factors, and the diagnosis can be delayed, increasing the risk of late presentation and recurrence. Thus, the clinicians should have a strong suspicion for persistent oral lesions regardless of smoking or drinking history. Future studies need to focus on standardizing definitions of NSND populations, clarifying causative pathways and developing focused prevention, surveillance and targeted therapy options. NSND OSCC should be considered not only as the absence of conventional exposures, but also as a biologically relevant disease subset requiring dedicated diagnostic awareness and customized management strategies.

## Figures and Tables

**Table 1:** Comparative characteristics of traditional (tobacco- and alcohol-related) and nonsmoking, nondrinking (NSND) oral squamous cell carcinoma.

Feature	Traditional OSCC (Smokers/Drinkers)	NSND OSCC
Primary risk factors	Tobacco, alcohol, betel quid, area nut [1,2,3]	Multifactorial: chronic mechanical irritation, microbiome dysbiosis, genetic susceptibility, metabolic and hormonal factors [3,11,12,13,14]
Epidemiological trend	Stabilizing or decreasing in regions with reduced tobacco use [4,19]	Increasing incidence, especially in developed countries [4,5,6,7]
Sex distribution	Predominantly male [8,19]	Predominantly female (≈65–77%) [8,9,10]
Age distribution	Peak incidence in 6th–7th decades [8]	Bimodal: younger (<45 years) and elderly (>70 years) [8,10,20]
Common anatomical sites	Floor of mouth, ventrolateral tongue [2,8]	Lateral tongue, gingiva, buccal mucosa [11,16,20]
Etiopathogenesis	Carcinogen-induced DNA damage and mutagenesis [1,2]	Multifactorial: inflammation, immune dysregulation, microbiome alterations, genetic and epigenetic mechanisms [1,3,16]
Viral involvement	Limited role in OSCC [21]	Minimal HPV role; possible EBV/HSV (“hit-and-run”) mechanisms [5,7,22,23]
Microbiome profile	Less clearly defined [7]	Enrichment of periodontal pathogens (*F. nucleatum*, *P. gingivalis*) [3,24]
Clinical suspicion	High due to recognized risk profile [15]	Often low → delayed diagnosis [15,16]
Tumor microenvironment	Variable immune response [16]	Increased TILs and PD-L1 expression [6,14,22,25]
Prognosis	Stage-dependent, variable [8,18]	Heterogeneous; favorable in some cohorts, worse in younger patients [8,18,24]
Recurrence/second primary tumors	Present, often linked to continued exposure [8]	Increased risk in certain subgroups [1,6,8,26]
Prevention strategies	Tobacco/alcohol cessation [1,2]	Broader risk assessment: oral hygiene, chronic trauma, systemic factors [11,13,14]

**Table 2:** Comparison of traditional and non-traditional risk factors in OSCC.

Aspect	Traditional Risk Factors (Tobacco/Alcohol-Related OSCC)	Non-Traditional Risk Factors (NSND OSCC)
Demographics	Typically affects older males, mainly in the sixth and seventh decades [1,2,4,19].	Shows female predominance, often 65–76.9% of cases, with a bimodal age distribution involving younger patients under 45 years and older patients over 70 years [1,6,8,9,10,19].
Tumor sites	More commonly associated with the floor of mouth [2,8,11,16,20].	More commonly affects the lateral tongue, gingiva or alveolar ridge, and buccal mucosa, while floor-of-mouth involvement is less common [6,10,11,16,19,20,24].
Prevalence trends	Historically the dominant OSCC phenotype and closely linked to tobacco and alcohol exposure, although its relative predominance is declining as tobacco exposure decreases [1,2,4,5,7,19].	Represents an increasing proportion of OSCC cases, accounting for approximately 15–35% in many cohorts, particularly in developed countries [6,8,9,10,25].
Main etiologic profile	Tobacco and alcohol are the major established carcinogenic exposures, with additional contribution from betel quid and area nut in endemic regions [1,2,3,4,9].	Etiology is multifactorial and includes chronic mechanical irritation, oral microbiome dysbiosis, genetic susceptibility, hormonal and metabolic factors, chronic inflammation, poor oral hygiene, and limited or alternative viral mechanisms. [3,5,11,12,13,14,16,17,18,22,29]
Clinical implication	Clinical suspicion may be higher because patients fit the classic behavioral risk profile of OSCC [1,2,19].	Diagnosis may be delayed because patients often lack conventional behavioral risk factors, and this subgroup may show higher recurrence and second primary tumor risk in some cohorts [1,6,8,16,26].

## Data Availability

No new data were created or analyzed in this study. Data sharing is not applicable to this article.

## Abbreviations

CMI	Chronic Mechanical Irritation
DSS	Disease-Specific Survival
EBV	Epstein–Barr Virus
FA	Fanconi Anemia
HNSCC	Head and Neck Squamous Cell Carcinoma
HPV	Human Papillomavirus
HSV	Herpes Simplex Virus
LMP-1	Latent Membrane Protein 1
NSND	Nonsmoking, Nondrinking/Nonsmokers and Nondrinkers
OPMD	Oral Potentially Malignant Disorder
OPSCC	Oropharyngeal Squamous Cell Carcinoma
OS	Overall Survival
OSCC	Oral Squamous Cell Carcinoma
PD-1	Programmed Death-1
PD-L1	Programmed Death-Ligand 1
PNI	Perineural Invasion
POI	Pattern of Invasion
SEER	Surveillance, Epidemiology, and End Results
SPT	Second Primary Tumor
TIL	Tumor-Infiltrating Lymphocyte
